# Long-term evolution of Valsalva retinopathy: a case series

**DOI:** 10.1186/1752-1947-6-346

**Published:** 2012-10-10

**Authors:** Miriam García Fernández, Joaquín Castro Navarro, Carmen González Castaño

**Affiliations:** 1 Department of Ophthalmology, Central University Hospital of Asturias, C/Dionisio Ridruejo, nº5, 11ºD, CP: 33007, Oviedo, Asturias, Spain

## Abstract

**Introduction:**

Valsalva retinopathy may occur as a sudden, dramatic loss of central vision due to the premacular location of the haemorrhage. It has been described in different clinical settings, and there are several options for its treatment.

**Case presentations:**

We present the cases of six patients with sudden visual acuity loss caused by Valsalva retinopathy, treated in our hospital in the last ten years. Case 1 involves a 32-year-old Caucasian man with a unilateral premacular haemorrhage after vomiting. A neodymium-doped yttrium aluminium garnet laser was used due to sufficient depth of the haemorrhage pocket, but it was unsuccessful. Instead, 20G pars plana vitrectomy was performed with excellent visual recuperation (visual acuity:1.0). Case 2 was of a 36-year-old Caucasian woman with Valsalva retinopathy after vomiting during pregnancy. A neodymium-doped yttrium aluminium garnet laser was also insufficient due to the coagulated blood. After labour, 23G pars plana vitrectomy was performed, and her final visual acuity was 1.0. Case 3 involved a 52-year-old Caucasian man with premacular bleeding due to vomiting after general anaesthesia. The haemorrhage did not resolve spontaneously, so 23G pars plana vitrectomy was performed, with excellent visual outcomes (visual acuity:1.0). Case 4 was a 24-year-old Caucasian man with a macular haemorrhage after thoracic trauma. He was observed over four weeks, after which we performed 23G pars plana vitrectomy, with complete visual restoration (visual acuity:1.0). Case 5 involved a 28-year-old man who developed a premacular bleed after vigorous dancing. After a period of observation, 23G pars plana vitrectomy was performed. A retinal break with a small haemorrhage around the break occurred, related to the peribulbar anaesthesia manoeuvers, but was resolved successfully. His final visual acuity was 1.0. Case 6 was a 22-year-old Caucasian woman who developed a premacular haemorrhage after weightlifting. Conservative management was performed due to the small size of her haemorrhage. It resolved spontaneously within one month, and her final visual acuity was 1.0.

**Conclusion:**

Valsalva retinopathy is a rare condition that causes a sudden loss of visual acuity. In patients with too dense haemorrhage, the best option could be the vitrectomy, with excellent visual outcomes, although surgery is not free of risks.

## Introduction

Valsalva retinopathy is an uncommon disease which presents itself with sudden visual loss due to a premacular haemorrhage, caused by a rapid increase in intraocular venous pressure. It often occurs in healthy young adults as a result of a variety of clinical settings: intense aerobic exercise [1], heavy lifting, straining on the toilet, vomiting (such as in pregnancy) [2], coughing, labour [3], thoracoabdominal trauma [4] or even vigorous sexual activity [5]. In patients with a large and dense haemorrhage, spontaneous resorption of the blood entrapped in the subhyaloid or sub-inner limiting membrane space may take months, and may result in permanent visual impairment due to pigmentary macular changes, formation of epiretinal membranes or toxic damage to the retina caused by prolonged contact with haemoglobin and iron [6]**.** Different techniques have been used to treat premacular haemorrhage. These include puncturing the posterior hyaloid face with a neodymium-doped yttrium aluminium garnet (Nd-YAG) [7,8] or green argon laser, pneumatic displacement of the haemorrhage by an intravitreal injection of gas [9] with or without recombinant tissue plasminogen activator [10] and pars plana vitrectomy [6].

We report the cases of six patients with a premacular haemorrhage due to Valsalva retinopathy, secondary to different conditions. We present the therapeutic procedures used and their long-term outcomes together with a literature review on this topic.

## Case presentations

**Figure 1:**
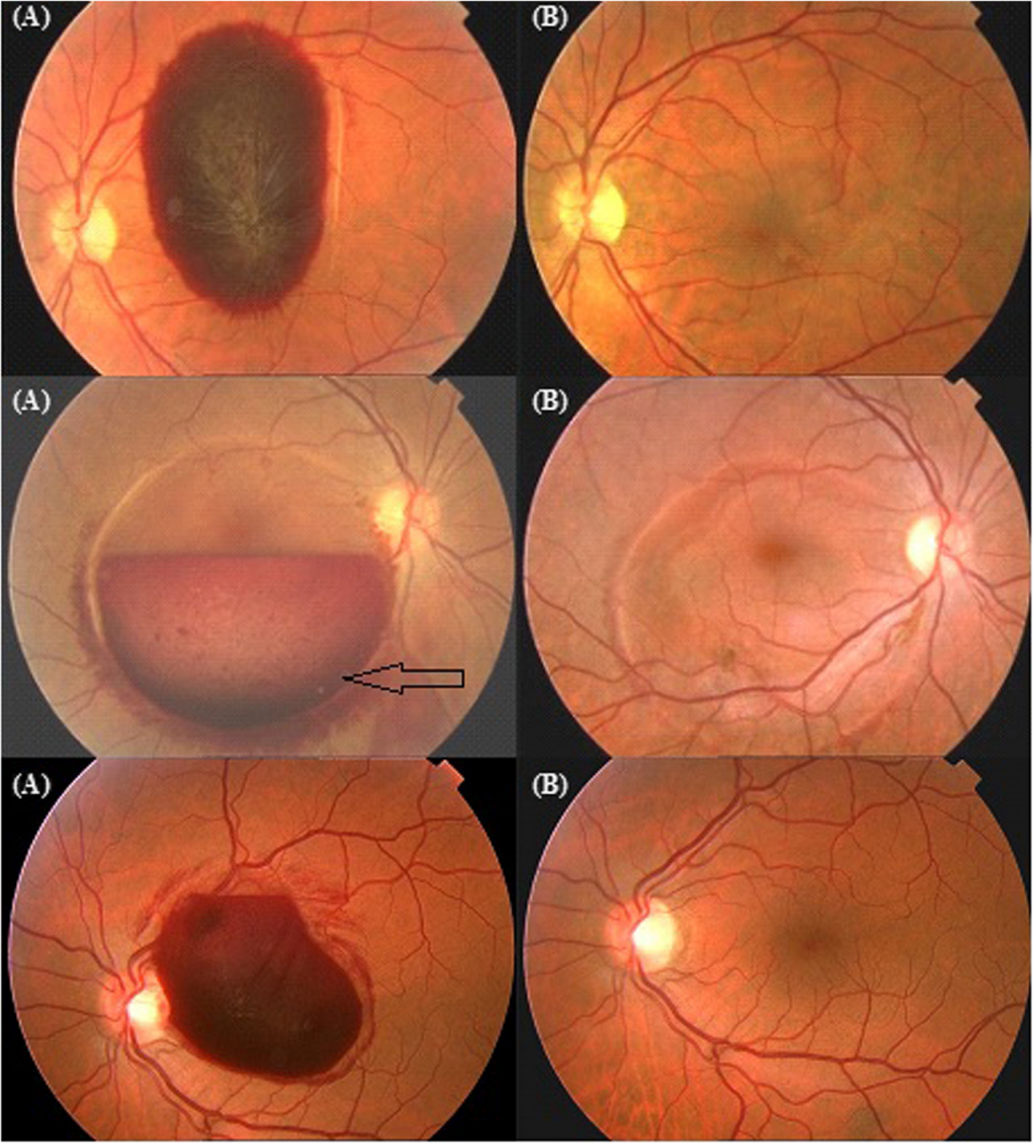
**Fundus photograph at (A) baseline examination and (B) after surgery in the patients whose aetiology was strong vomiting (cases 1, 2 and 3).** The black arrow shows the Nd-YAG laser treatment performed prior to the vitrectomy in case 2.

**Figure 2:**
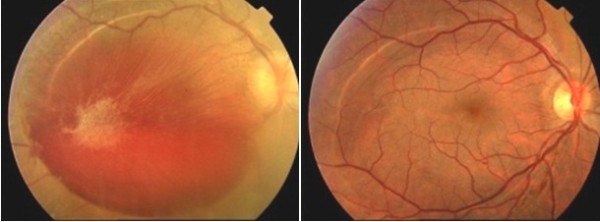
Fundus photograph prior to and after surgery in Valsalva retinopathy due to thoracoabdominal trauma (case 4).

**Figure 3:**
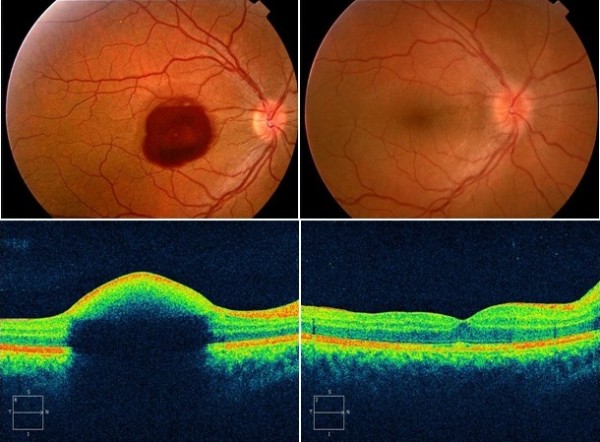
Fundus photograph and OCT prior to and after surgery in Valsalva retinopathy due to vigorous dancing (case 5).

**Figure 4:**
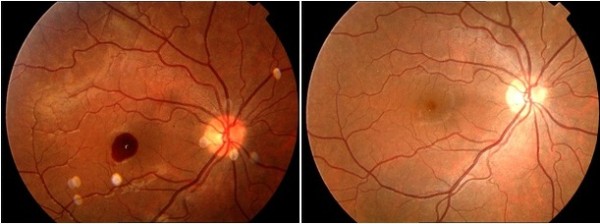
Fundus photograph at baseline and final examination of a Valsalva retinopathy due to weightlifting, which resolved spontaneously (case 6).

**Figure 5:**
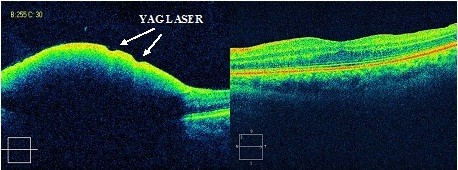
Optical coherence tomography prior to and after vitrectomy in a patient previously treated with Nd-YAG laser, without success (case 2).

Individual patient data at baseline and final examination are presented in Table 1. Fundus photographs are presented for all patients in Figures 1, 2, 3 and 4. Figure 5 shows the optical coherence tomography image for the patient from case 2.

**Table 1:** Patient data at baseline and final examination

**Patient**	**Age (years)**	**Aetiology**	**Initial BCVA (Snellen)**	**Treatment**	**Final BCVA (Snellen)**	**Follow-up (months)**
1	32	Strong vomiting	0.02	20G VTM	1.0	59
2	36	Vomiting (pregnancy)	0.1	23G VTM	1.0	44
3	52	Vomiting (general anaesthesia)	0.02	23G VTM	1.0	30
4	24	Trauma	0.05	23G VTM	1.0	116
5	28	Dancing	0.02	23G VTM	1.0	26
6	22	Weightlifting	0.20	Observation	1.0	39

BCVA: best corrected visual acuity; VTM: vitrectomy.

### Case 1

A 32-year-old Caucasian man developed a unilateral premacular haemorrhage after strong vomiting associated with a digestive disease. Immediately after vomiting, he noticed a sudden decrease in vision. His visual acuity (VA) at first examination was 0.02 (decimal notation). We tried Nd:YAG laser treatment because the haemorrhage pocket was deep enough to prevent damage to the retina. This treatment was unsuccessful, probably because of the coagulated trapped blood. So, we performed 20G three-port pars plana vitrectomy. A posterior vitreous detachment was created because it was not already present. The internal limiting membrane (ILM) was released and the haemorrhage was cleaned. The sub-ILM localization of the haemorrhage was confirmed during the vitrectomy.

His VA improved one day after surgery, and was 10 out of 10 one week later. It remained unchanged during the follow-up period of 59 months.

### Case 2

A 36-year-old Caucasian woman presented with unilateral sudden visual loss after vomiting during the eighth month of her pregnancy. Her VA at first examination was 0.1. A huge premacular haemorrhage was observed during a fundus examination. As in the previous case, we first tried Nd:YAG laser treatment because our patient could not undergo vitrectomy owing to the risk to the foetus. The laser treatment was unsuccessful.

After labour, a 23G pars plana vitrectomy was performed, following the same technique previously described. The sub-ILM localization of the haemorrhage was also confirmed during the vitrectomy.

Her VA improved 24 hours after surgery, and was 10 out of 10 seven days later. It remained unchanged during the follow-up period of 44 months.

### Case 3

A 52-year-old Caucasian man complained of a decrease in vision after strong vomiting associated with general anaesthesia for cervical lipoma surgery. He had developed a unilateral premacular haemorrhage. His VA at first visit was 0.02 (decimal notation).

We decided to observe the evolution of the haemorrhage over a period of four weeks, and no significant changes were observed in this period. We then performed 23G three-port pars plana vitrectomy. The surgical technique was the same as previously described. The sub-ILM localization of the haemorrhage was also confirmed during the vitrectomy.

His VA improved one day after surgery, and was 10 out of 10 six days later. It remained without changes during the follow-up period of 30 months.

### Case 4

A 24-year-old Caucasian man experienced a thoracoabdominal trauma secondary to a traffic accident. One week after the trauma, he was referred to our department for visual loss in his right eye.

At first examination, we found a huge premacular haemorrhage of six disc diameter in size. His VA was 0.05. Similar to previous cases, our patient was observed for four weeks, after which we decided to perform 23G three-port pars plana vitrectomy with the same technique described previously. Visual restoration was excellent and complete, with his VA being 10 out of 10 one week after surgery, and remaining stable during 116 months of follow-up.

### Case 5

A 28-year-old man complained of sudden visual loss in his right eye 24 hours after vigorous dancing. At first examination, we observed a premacular haemorrhage of two disk diameter in size, and a VA of 0.02. Clinical observation was performed for three weeks, after which he underwent a 23G vitrectomy, as described previously. The vitrectomy confirmed the sub-ILM localization of the haemorrhage, as in our other reported cases.

A retinal break with a small haemorrhage around the break, related to the peribulbar anaesthesia manoeuvers, was observed at the beginning of the vitrectomy. This complication was resolved successfully by applying an argon laser around the break and fluid-gas SF_6_ (sulphur hexafluoride) exchange at the end of the surgery. Four months later, our patient developed a cataract and underwent phacoemulsification and intraocular lens implantation without any complications. His VA after 26 months of follow-up was 1.0 (decimal notation).

### Case 6

A 22-year-old Caucasian woman came to our emergency room with a complaint of blurred vision in her right eye three hours after participating in weightlifting. She presented with a small haemorrhage in the macula, so we decided on conservative management. One month later, the haemorrhage resolved itself spontaneously and her VA recovered to 10 out of 10.

## Discussion

Haemorrhages of less than one disk diameter tend to spontaneously resolve in a short period of time and a conservative approach is generally justifiable, as happened in one of our patients (case 6). By contrast, the spontaneous resolution of large and dense haemorrhages, as we presented in five cases of this study, is very unlikely. Several types of treatment are available, such as drainage of the haemorrhage into the vitreous cavity with Nd-YAG laser hyaloidotomy [7,8,10], gas injection [11] with or without recombinant tissue plasminogen activator [9], classical pars plana vitrectomy [6] and non-vitrectomising vitreous surgery as reported by Wu *et al*. [12]. However, when the premacular blood is coagulated, drainage with YAG laser is not possible, as demonstrated in two of our patients. Moreover, the proximity to the retinal surface presents an additional problem when using the laser. Complications of Nd:YAG laser membranotomy include creating a macular hole or persistent premacular cavity, retinal detachment and epiretinal membrane formation [13,14]. Nd:YAG laser can be useful in the treatment of non-dense and non-coagulated premacular haemorrhages, and when the location of the blood is subhyaloid, but not when there is blood under the ILM.

However, we should assume the possibility of blood localization errors even when using advanced retinal imaging techniques such as high resolution optical coherence tomography.

## Conclusion

We suggest that vitrectomy is more effective and safer than other treatment modalities in the management of patients with dense premacular haemorrhages and insufficient spontaneous reabsorption. However, surgery is not free of risks, and complications such as cataract or retinal breaks, as was observed in one of our patients (case 5), may occur.

## Consent

Oral and written informed consent was obtained from all the patients for publication of this case report and accompanying images. A copy of the written consent is available for review by the Editor-in-Chief of this journal.

## Competing interests

The authors declare that they have no competing interests.

## Authors’ contributions

JCN and CGC photographed and interpreted the pathologic findings, and performed all the surgeries. MGF drafted the article, and analysed and interpreted the patient data. All authors have made substantive intellectual contributions to this study and to the manuscript and have read and approved the final manuscript.
